# Effects of serelaxin in acute heart failure patients with renal impairment: results from RELAX-AHF

**DOI:** 10.1007/s00392-016-0979-8

**Published:** 2016-03-26

**Authors:** Licette C. Y. Liu, Adriaan A. Voors, John R. Teerlink, Gad Cotter, Beth A. Davison, G. Michael Felker, Gerasimos Filippatos, Yakuan Chen, Barry H. Greenberg, Piotr Ponikowski, Peter S. Pang, Margaret F. Prescott, Tsushung A. Hua, Thomas M. Severin, Marco Metra

**Affiliations:** 1 Department of Cardiology, University Medical Center Groningen, Hanzeplein 1, 9713 GZ Groningen, The Netherlands; 2 San Francisco Veterans Affairs Medical Center, University of California, San Francisco, CA USA; 3 Momentum Research, Durham, NC USA; 4 Duke University School of Medicine, Duke Heart Center, Durham, NC USA; 5 School of Medicine, Attikon University Hospital, National and Kapodistrian University of Athens, Athens, Greece; 6 Columbia University Medical Center, New York, NY USA; 7 University of California at San Diego, San Diego, CA USA; 8 Clinical Military Hospital, Medical University, Wroclaw, Poland; 9 Indiana University School of Medicine, Indianapolis, IN USA; 10 Novartis Pharma, Basel, Switzerland; 11 University of Brescia, Brescia, Italy

**Keywords:** Serelaxin, Acute heart failure, Renal function, Renal impairment, Number needed to treat

## Abstract

**Background:**

Serelaxin showed beneficial effects on clinical outcome and trajectories of renal markers in patients with acute heart failure. We aimed to study the interaction between renal function and the treatment effect of serelaxin.

**Methods:**

In the current post hoc analysis of the RELAX-AHF trial, we included all patients with available estimated glomerular filtration rate (eGFR) at baseline (*n* = 1132). Renal impairment was defined as an eGFR <60 ml/min/1.73 m^2^ estimated by creatinine.

**Results:**

817 (72.2 %) patients had a baseline eGFR <60 ml/min/1.73 m^2^. In placebo-treated patients, baseline renal impairment was related to a higher 180 day cardiovascular (HR 3.12, 95 % CI 1.33–7.30) and all-cause mortality (HR 2.81, 95 % CI 1.34–5.89). However, in serelaxin-treated patients, the risk of cardiovascular and all-cause mortality was less pronounced (HR 1.19, 95 % CI 0.54 –2.64; *p* for interaction = 0.106, and HR 1.15 95 % CI 0.56–2.34 respectively; *p* for interaction = 0.088). In patients with renal impairment, treatment with serelaxin resulted in a more pronounced all-cause mortality reduction (HR 0.53, 95 % CI 0.34–0.83), compared with patients without renal impairment (HR 1.30, 95 % CI 0.51–3.29).

**Conclusion:**

Renal dysfunction was associated with higher cardiovascular and all-cause mortality in placebo-treated patients, but not in serelaxin-treated patients. The observed reduction in (cardiovascular) mortality in RELAX-AHF was more pronounced in patients with renal dysfunction. These observations need to be confirmed in the ongoing RELAX-AHF-2 trial.

## Introduction

Renal dysfunction and worsening of renal function are frequently found in acute heart failure patients and are both associated with prolonged hospital stay, and higher risk of rehospitalization and mortality [1–7]. The Relaxin in Acute Heart Failure (RELAX-AHF) trial studied the effects of serelaxin, a novel recombinant of the naturally occurring human relaxin-2 vasoactive peptide, intravenously administrated for 48 h in acute heart failure patients [8]. The RELAX-AHF demonstrated that treatment with serelaxin resulted in beneficial effects on dyspnea, compared with placebo. Interestingly, while only administrated for 48 h, serelaxin resulted in a significant reduction of cardiovascular (CV) and all-cause death up to 180 days [8]. A post hoc analysis demonstrated that patients treated with serelaxin had lower increases of markers of end-organ damage, including the renal markers creatinine and cystatin C, during hospitalization [9]. These observations suggested that serelaxin may have beneficial effects on renal function in patients with acute heart failure. In fact, in studies investigating the (renal) hemodynamic effects of serelaxin in healthy volunteers, chronic heart failure, and acute heart failure, and a study investigating the antifibrotic effects of serelaxin in patients with diffuse scleroderma both found that serelaxin improved creatinine clearance and renal blood flow [10–13]. The post hoc analysis on the effect of serelaxin on biomarkers of end-organ damage was then followed by a large subgroup analysis, studying the effect of serelaxin in several subgroups based on clinical characteristics on the endpoints: dyspnea relief by visual analog scales (VAS) AUC to day 5, the composite of CV death/rehospitalization for heart failure or renal failure through day 60, and CV death through day 180. Overall, no evident differences in the effects of serelaxin vs. placebo were observed across the subgroups. However, there were some suggestions that serelaxin may have had a greater treatment effect in patients with impaired renal function at baseline [14, 15]. In the present paper, we aimed to investigate whether serelaxin mitigates the association between renal impairment and poor clinical outcome in acute heart failure. In addition, we aimed to study the detailed effects of serelaxin on other outcomes including total dose of diuretics, worsening heart failure, hospital stay, days alive out of the hospital, CV death through day 60 and all-cause mortality through day 180 in patients with renal impairment at baseline.

## Methods

### Patient population

For the present analyses, we studied all patients with available estimated glomerular filtration rate (eGFR) at baseline enrolled in the RELAX-AHF trial. The methods and main results of the study have been published in detail elsewhere [8, 16]. Between October 2009 and February 2012, 1161 acute heart failure patients were randomly assigned within 16 h of presentation to one of the two treatment groups in a 1:1 ratio (serelaxin 30 μg/kg per day or placebo). Eligible patients were adult male or female, hospitalized for acute heart failure, defined as dyspnea at rest or with minimal exertion, pulmonary congestion on chest radiograph and elevated natriuretic peptide levels [brain natriuretic peptide (BNP) or N-terminal proBNP (NT-proBNP) levels ≥350 or ≥1400 pg/mL, respectively], requiring at least 40 mg intravenous furosemide or its equivalent. Further, patients had to have a systolic blood pressure of >125 mm Hg and mild to moderate renal function (estimated creatinine clearance of 30–75 ml/min/1.73 m^2^). Key exclusion criteria included signs of active infection, significant pulmonary or valvular disease, acute heart failure due to significant arrhythmias, acute coronary syndrome 45 days prior screening or a troponin level >3 times the level diagnostic of myocardial infarction, and treatment with any other intravenous therapy (except intravenous nitrate at dose of <0.1 mg/kg in patients with systolic blood pressure >150 mmHg). The RELAX-AHF trial was conducted under International Committee on Harmonization Good Clinical Practices and applicable country and local regulations, and approved by the Ethics Committee of each participating site. All patients provided written informed consent. The RELAX-AHF trial is registered at Clinicaltrials.gov, number NCT00520806.

### Study procedures

Patients were randomized within 16 h of presentation to one of the two intervention groups receiving either serelaxin 30 μg/kg per day or placebo intravenously for up to 48 h continuously. Physical examinations were done, and patient reported changes in dyspnea severity and general well-being were measured at baseline (by VAS) and at 6, 12, 24 and 48 h, and then daily to day 5 (by Likert and VAS). The onset of worsening heart failure (WHF), worsening of signs or symptoms of heart failure necessitating treatment intensification, was evaluated daily to day 5 and day 14. Patients who died by day 5 without a prior WHF event were assumed to have had WHF on the day of death. Patients were followed on days 14, 60 and 180. Non-serious adverse events (AE) were collected through day 5, while serious AE (SAE) were reported through day 14.

### Renal function

eGFR was calculated by the simplified Modification of Diet in Renal Disease formula based on creatinine [17–19]. Renal impairment was defined as eGFR <60 ml/min/1.73 m^2^. Creatinine was measured in serum using the Roche CREA plus enzymatic assay (Roche Diagnostics, Mannheim, Germany).

### Treatment effect of serelaxin in patients with renal impairment

We studied the treatment effect of serelaxin in all patients with available eGFR at baseline and patients with an eGFR under and equal or above 60 ml/min/1.73 m^2^ at baseline on the following clinical outcomes: moderate or marked dyspnea relief by Likert scale at 6, 14 and 24 h, total dose of intravenous loop diuretic to day 5, total dose of oral loop diuretic to day 5, worsening heart failure through day 5 and day 14, length of initial hospital stay (days), length of stay in intensive care unit (ICU)/coronary care unit (CCU) (days), days alive out of hospital through day 60, CV death through day 60 and all-cause mortality through day 180. In addition, we estimated the number needed to treat (NNT) to prevent one CV death and to prevent one death from any cause in patients with renal impairment and the overall study population.

### Statistical analysis

For baseline characteristics, mean (SD), or geometric mean (95 % CI) if log transformed, was reported for continuous variables; and frequencies and proportions (%) for categorical variables (all for patients with non-missing values of the variable of interest). Baseline characteristics of patients with and without renal impairment were compared using *t* tests (assuming equal or unequal variances, as appropriate) for continuous variables and Chi squared or Fisher’s Exact tests for categorical variables.

To investigate the association between renal impairment and clinical outcomes, and the possible interactions of serelaxin and renal impairment effects on clinical outcomes, for continuous outcomes we constructed multiple linear regression models including the main effects of renal impairment, treatment group, and their interaction; least square means and mean differences (with 95 % CIs), and *p* values for the interaction of treatment with renal impairment are presented. For binary endpoints, we generated two-by-two contingency tables to report *n* (%) and odds ratios (with 95 % CIs), and used logistic regression to obtain *p* values for the interaction of treatment with renal impairment. For time-to-event endpoints, we present the number of events, Kaplan–Meier estimates of the event rates, and hazard ratios (with 95 % CIs) and *p* values for the interaction of treatment with renal impairment from Cox proportional hazards models. The number needed to treat (NNT) was estimated based on Kaplan–Meier survival probabilities at day 180 by treatment group for the renal impairment subgroup and the total population [20].

## Results

Baseline characteristics of patients enrolled in RELAX-AHF trial according to eGFR are presented in Table 1. The subgroup eGFR <60 ml/min/1.73 m^2^ included 817 patients, the subgroup eGFR ≥60 ml/min/1.73 m^2^ included 315 patients. Patients with an eGFR <60 ml/min/1.73 m^2^ were older, and were more likely to have a history of hypertension, hyperlipidemia, diabetes mellitus, and ischemic heart disease.

**Table 1:** Baseline characteristics according estimated glomerular filtration rate

Variables	eGFR <60 ml/min/1.73 m^2^ (*N* = 817)^a^	eGFR ≥60 ml/min/1.73 m^2^ (*N* = 315)^a^	*p* value^b^
Demographics and heart failure characteristics			
Age (years)	73.3 (10.6)	68.8 (12.1)	<0.001^x^
Male	505 (61.8)	200 (63.5)	0.601^†^
White/Caucasian	772 (94.5)	301 (95.6)	0.471^†^
Left ventricular ejection fraction (%)	39.2 (14.6)	37.0 (14.3)	0.027^*^
Ischemic heart disease	443 (54.2)	146 (46.3)	0.018^†^
NYHA class (I/II/III/IV) 30 days before admission			0.130^†^
I	216 (26.7)	100 (31.9)	
II	210 (26.0)	84 (26.8)	
III	289 (35.7)	90 (28.8)	
IV	94 (11.6)	39 (12.5)	
Clinical signs			
Body mass index (kg/m^2^)	29.3 (5.7)	29.2 (5.8)	0.918^*^
Syst. blood pressure (mmHg)	142.4 (16.7)	141.6 (15.8)	0.507^*^
Diast. blood pressure (mmHg)	78.4 (14.3)	80.7 (13.8)	0.012^*^
Heart rate, beat per minute	78.9 (14.7)	82.1 (15.2)	0.002^*^
Serelaxin administration (%)	409 (50.1)	155 (49.2)	0.797^†^
Medical history			
Hypertension	720 (88.1)	258 (81.9)	0.006^†^
Hyperlipidemia	454 (55.6)	146 (46.3)	0.005^†^
Diabetes mellitus	414 (50.7)	125 (39.7)	0.001^†^
Cigarette smoking	96 (11.8)	51 (16.2)	0.046^†^
Stroke or other cerebrovascular event	111 (13.6)	42 (13.3)	0.911^†^
Peripheral vascular disease	115 (14.1)	35 (11.1)	0.187^†^
Asthma, bronchitis, or COPD	135 (16.5)	42 (13.3)	0.185^†^
Atrial fibrillation at screening	344 (42.2)	122 (38.7)	0.287^†^
History of Atrial fibrillation or flutter	439 (53.7)	149 (47.3)	0.052^†^
History of CRT or ICD procedures	228 (27.9)	61 (19.4)	0.003^†^
Myocardial infarction	291 (35.6)	101 (32.1)	0.260^†^
Depression	39 (4.8)	19 (6.0)	0.390^†^
Baseline laboratory			
Hemoglobin (g/dL)	12.58 (1.89)	13.31 (1.67)	<0.001^x^
Sodium (mmol/L)	140.84 (3.61)	140.80 (3.53)	0.853^*^
Potassium (mmol/L)	4.33 (0.64)	4.12 (0.59)	<0.001^*^
Uric acid (μmol/L)	490.8 (135.8)	436.9 (128.5)	<0.001^*^
BUN (mmol/L)	10.82 (4.02)	7.09 (2.53)	<0.001^x^
Cystatine C (mg/L)^c^	1.60 (1.57, 1.63)	1.14 (1.11, 1.17)	<0.001^*^
NT-proBNP (ng/L)^c^	5567 (5236, 5920)	3883 (3521, 4281)	<0.001^*^
hsTnT (ng/L)^c^	0.037 (0.035, 0.040)	0.029 (0.026, 0.032)	<0.001^*^
Medication (day 0)			
ACE inhibitor	431 (52.8)	189 (60.0)	0.028^†^
ACEi or ARBs	546 (66.8)	227 (72.1)	0.090^†^
Angiotensin-receptor blocker	136 (16.6)	46 (14.6)	0.402^†^
Beta-blocker	565 (69.2)	214 (67.9)	0.692^†^
Aldosterone antagonist	252 (30.8)	105 (33.3)	0.419^†^
Digoxin	167 (20.4)	60 (19.0)	0.600^†^

^a^Mean (SD), or geometric mean (95 % CI) if log transformed, for continuous variables; *n* (%) for categorical variables (% based on total number of patients with non-missing values of the variable of interest)

^b^ *P* value is based on * *t* test, ^†^ Chi squared test, ^‡^ Fisher’s Exact test, or the ^x^ Satterthwaite method due to unequal variances in comparison groups. Statistical tests are not adjusted for multiple comparisons

^c^The following ‘Baseline labs’ variables have been log transformed: hsTnT, NT-proBNP, Cystatine C

### Association between renal impairment and clinical outcomes

**Fig. 1:**
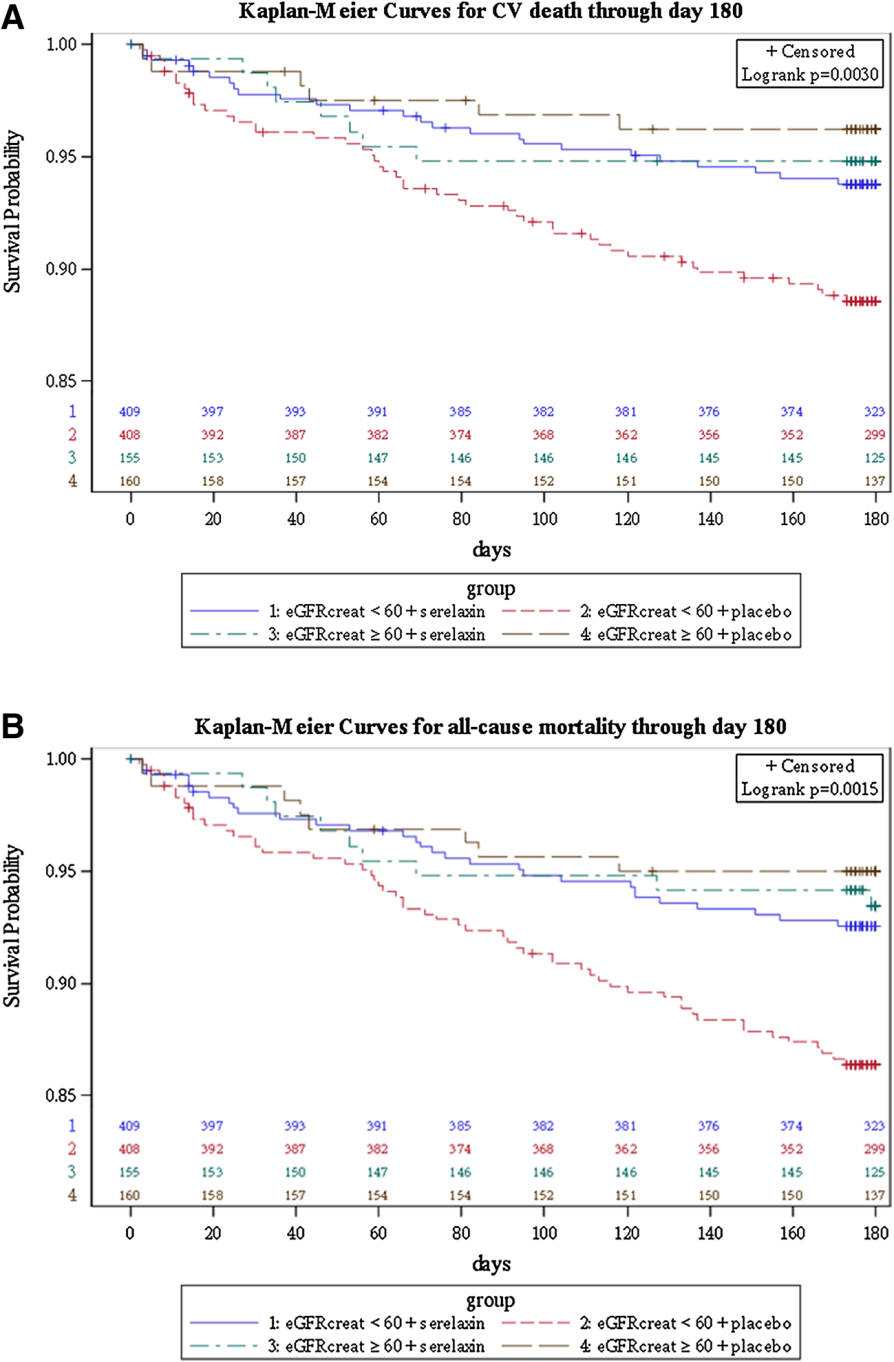
Kaplan–Meier curves for cardiovascular (CV) death through day 180 (**a** *upper panel*) and all-cause death through day 180 (**b** *lower panel*) according to eGFR. *eGFR* estimated glomerular filtration rate

**Fig. 2:**
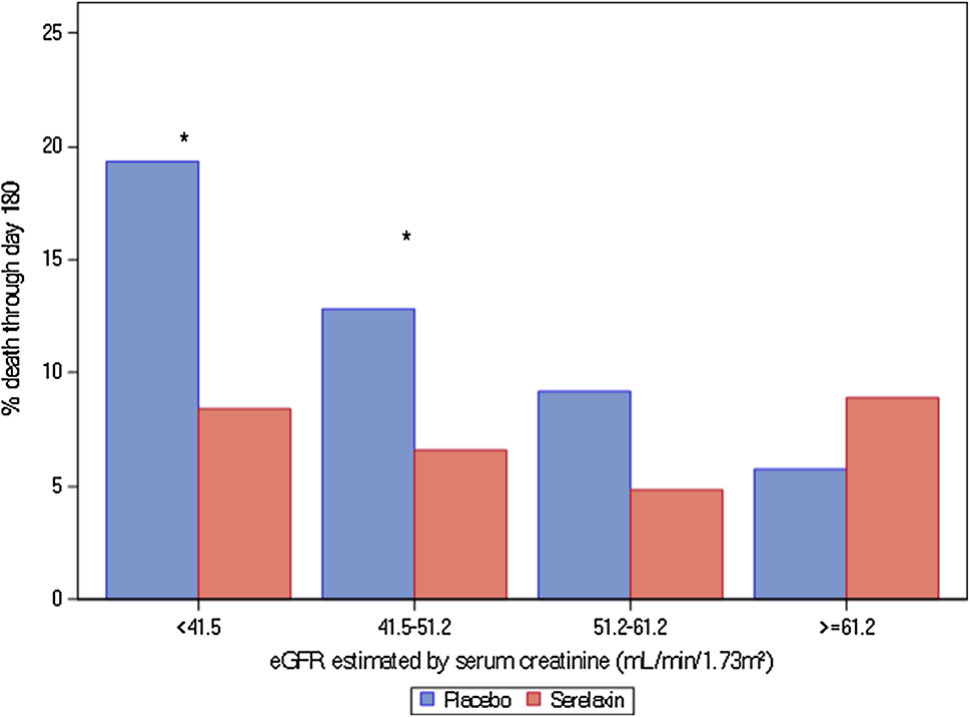
Percentage of death through day 180 in subgroups according to estimated glomerular filtration (eGFR) rate in acute heart failure patients treated with placebo (*blue*) or serelaxin (*red*). (1) *p < 0.05; (2) eGFR interval 41.5–51.2 stands for ≥41.5 and <51.2; 51.2–61.2 stands for ≥51.2 and <61.2

The association between renal impairment and clinical outcomes in the overall study population, the serelaxin group and the placebo group are presented in Table 2. In the overall study population, renal impairment was significantly associated with an increased risk of 180 day CV (HR 2.00, 95 % CI 1.13–3.54, *p* for log rank test = 0.016) and all-cause mortality (HR 1.86, 95 % CI 1.12–3.10, *p* for log rank test = 0.015). We found no significant interactions between the association of renal impairment, poor clinical outcome and study treatment on any outcomes. However, a trend towards an attenuated association between renal impairment and mortality was observed in patients treated with serelaxin. Patients with an eGFR <60 ml/min/1.73m^2^ treated with placebo group, tended to have a higher risk of CV death through day 60 (HR 2.20, 95 % CI 0.76–6.38) compared with patients with an eGFR <60 ml/min/1.73m^2^ treated with serelaxin (HR 0.65, 95 % CI 0.26–1.66, *p* for interaction = 0.093). In the placebo group, eGFR <60 ml/min/1.73m^2^ was associated with CV mortality through day 180 (HR 3.12, 95 % CI 1.33–7.30, while in the serelaxin group, a tendency towards attenuation of this association was observed (HR 1.19, 95 % CI 0.54–2.64, *p* for interaction = 0.106). In the placebo group, eGFR <60 ml/min/1.73m^2^ was associated with all-cause mortality through day 180 (HR 2.81, 95 % CI 1.34–5.89), while in the serelaxin group, a trend towards attenuation of this association was observed (HR 1.15, 95 % CI 0.56–2.34, *p* for interaction = 0.088). Figure 1 presents the survival probabilities for patients with and without renal impairment treated with serelaxin or placebo. Patients with eGFR <60 ml/min/1.73 m^2^ treated with placebo, had worse survival curves compared to both patients with eGFR <60 ml/min/1.73 m^2^ treated with serelaxin and patients with normal renal function regardless of their study treatment. Figure 2 summarizes the all-cause mortality rate through day 180 in both treatment groups by baseline eGFR, showing a greater treatment effect of serelaxin as eGFR is declining.

**Table 2:** Association between renal impairment and clinical outcomes in the all patients, serelaxin group and placebo group

	All patients (*n* = 1132)^a^	Serelaxin (*n* = 564)^a^	Placebo (*n* = 568)^a^	Interaction *p* value^b^
Dyspnea relief by VAS AUC to day 5	−139.39 (−510.72, 231.95)	−30.03 (−556.94, 496.88)	−253.80 (−774.89, 267.28)	0.554
Dyspnea relief by Likert scale at 6, 12 and 24 h	0.99 (0.74, 1.33)	1.14 (0.74, 1.73)	0.87 (0.58, 1.32)	0.384
Worsening heart failure through day 5	1.16 (0.75, 1.80)	1.48 (0.68, 3.22)	1.04 (0.61, 1.76)	0.460
Worsening heart failure through day 14	1.28 (0.88, 1.86)	1.70 (0.91, 3.19)	1.07 (0.67, 1.71)	0.246
CV death/re-hospitalization for HF/RF through day 60 (days)	1.39 (0.94, 2.06)	1.14 (0.67, 1.95)	1.72 (0.96, 3.08)	0.311
CV death through day 60 (days)	1.21 (0.61, 2.38)	0.65 (0.26, 1.66)	2.20 (0.76, 6.38)	0.093
CV death through day 180	2.00 (1.13, 3.54)	1.19 (0.54, 2.64)	3.12 (1.33, 7.30)	0.106
All-cause mortality day 180	1.86 (1.12, 3.10)	1.15 (0.56, 2.34)	2.81 (1.34, 5.89)	0.088

^a^Effect of eGFR <60 mL/min/1.73m^2^ is estimated: LS mean difference (95 % CI) for continuous endpoints, odds ratio (95 % CI) from 2 × 2 contingency table for binary endpoints, and hazard ratio (95 % CI) from Cox proportional hazards model for time-to-event endpoints

^b^Interaction *p* values are based on tests of treatment-by-eGFR interaction. Interaction *p* values are obtained using Cox regression for time-to-event endpoints, logistic regression for binary endpoints, and multiple linear regression for continuous endpoints

### Effects of serelaxin in acute heart failure patients with renal impairment

We compared the effect of serelaxin on clinical outcomes in patients with and without renal impairment. The treatment effect of serelaxin in the overall study population is also summarized in Table 3. A trend towards a higher CV death toll through day 60 reduction by serelaxin was observed in patients with renal impairment compared with patients without renal impairment (HR 0.54 vs. 1.82, *p* for interaction = 0.093). A similar trend was observed for 180 day all-cause mortality, showing the beneficial effects of serelaxin in patients with an eGFR under 60 ml/min/1.73 m^2^ compared with patients with an eGFR equal or above 60 ml/min/1.73 m^2^ (HR 0.53 vs. 1.30, *p* for interaction = 0.088). Patients treated with serelaxin tended to require less intravenous (IV) diuretics but more oral diuretics to day 5, had a shorter stay at the hospital and ICU/CCU, and had more days alive and out of the hospital through day 60. These observations were more pronounced in the eGFR <60 ml/min/1.73 m^2^ subgroup compared with the eGFR ≥60 ml/min/1.73 m^2^ subgroup although these interactions were not statistically significant (*p* for interaction IV diuretic = 0.608, *p* for interaction oral diuretic = 0.849, *p* for interaction initial hospital stay = 0.347, *p* for interaction length of stay in ICU/CCU = 0.444, *p* for interaction days alive and out of the hospital through day 60 = 0.422; Table 3).

**Table 3:** Treatment effect of serelaxin in all patients, eGFR <60 and eGFR ≥60 subgroup

	All patients (*n* = 1132)		eGFR <60 (*n* = 817)		eGFR ≥60 (*n* = 315)		Treatment effect of serelaxin			Interaction *p* value^c^
	Serelaxin (*n* = 564)^a^	Placebo (*n* = 568)^a^	Serelaxin (*n* = 409)^a^	Placebo (*n* = 408)^a^	Serelaxin (*n* = 155)^a^	Placebo (*n* = 160)^a^	All patients	eGFR <60^b^	eGFR ≥60^b^	
Dyspnea relief by Likert scale at 6, 12 and 24 h (number of patients, %)	152 (26.95)	149 (26.23)	113 (27.63)	104 (25.49)	39 (25.16)	45 (28.13)	1.04 (0.80, 1.35)	1.12 (0.82, 1.52)	0.86 (0.52, 1.42)	0.384
Total dose of IV diuretic to day 5 (mg)	161 (135, 187)	211 (185, 237)	170 (139, 201)	226 (195, 257)	136 (86, 187)	171 (122, 220)	−50 (−87, −13)	−56 (−100, −13)	−34 (−105, 36)	0.608
Total dose of oral diuretic to day 5 (mg)	193 (177, 209)	184 (168, 200)	197 (178, 216)	187 (168, 206)	183 (152, 213)	177 (147, 207)	9 (−14, 32)	10 (−16, 37)	5 (−38, 48)	0.849
Worsening heart failure through day 5	39 (6.94)	69 (12.15)	31 (7.60)	50 (12.25)	8 (5.19)	19 (11.88)	0.55 (0.37, 0.82)	0.60 (0.38, 0.94)	0.42 (0.18, 0.96)	0.460
Worsening heart failure through day 14	65 (11.58)	89 (15.68)	53 (13.01)	65 (15.95)	12 (7.79)	24 (15.00)	0.71 (0.52, 0.98)	0.79 (0.55, 1.13)	0.50 (0.25, 0.99)	0.246
Length of initial hospital stay (days)	9.66 (8.89, 10.43)	10.40 (9.64, 11.17)	9.55 (8.64, 10.45)	10.61 (9.71, 11.52)	9.95 (8.49, 11.42)	9.86 (8.42, 11.30)	−0.74 (−1.83, 0.34)	−1.07 (−2.34, 0.21)	0.09 (−1.96, 2.15)	0.347
Length of stay in ICU/CCU (days)	3.55 (2.97, 4.12)	3.80 (3.23, 4.38)	3.42 (2.74, 4.10)	3.88 (3.20, 4.56)	3.87 (2.76, 4.98)	3.61 (2.53, 4.70)	−0.26 (−1.07, 0.56)	−0.46 (−1.42, 0.51)	0.26 (−1.29, 1.81)	0.444
Days alive out of hospital through day 60 (days)	48.28 (47.31, 49.26)	47.74 (46.77, 48.71)	48.32 (47.17, 49.47)	47.42 (46.27, 48.57)	48.19 (46.32, 50.05)	48.55 (46.71, 50.39)	0.54 (−0.84, 1.92)	0.90 (−0.73, 2.52)	−0.36 (−2.98, 2.25)	0.422
CV death through day 60	19 (3.40)	26 (4.61)	12 (2.97)	22 (5.43)	7 (4.55)	4 (2.51)	0.73 (0.41, 1.32)	0.54 (0.27, 1.09)	1.82 (0.53, 6.21)	0.093
All-cause mortality day 180	40 (7.19)	63 (11.18)	30 (7.43)	55 (13.61)	10 (6.56)	8 (5.01)	0.63 (0.42, 0.94)	0.53 (0.34, 0.83)	1.30 (0.51, 3.29)	0.088

^a^LS Mean (95 % CI) for continuous endpoints, *n* (KM %) for time-to-event endpoints, and *n* (%) for binary endpoints

^b^LS mean difference (95 % CI) for continuous endpoints, odds ratio (65 % CI) from 2 × 2 contingency table for binary endpoints, and hazard ratio (95 % CI) from Cox proportional hazards model for time-to-event endpoints

^c^Interaction *p* values are based on test of treatment-by-eGFR interaction. Interaction *p* value estimates are obtained using Cox model for time-to-event endpoints, logistics regression for binary endpoints, and regression model for continuous endpoints

### Number needed to treat

The number of patients that needed to be treated with serelaxin to prevent one CV death through day 180 in the overall study population was 29. In the eGFR <60 ml/min/1.73 m^2^ subgroup, the number needed to treat (NNT) to prevent one CV death was 19. The number of patients that needed to be treated with serelaxin to prevent one all-cause death during a follow-up of 180 days in the overall study population was 25. In the eGFR <60 ml/min/1.73 m^2^ subgroup, the NNT to prevent one all-cause death was 16.

### Safety of serelaxin

The safety of serelaxin in the overall study population, and patients with and without renal impairment defined by eGFR are summarized in Table 4. In general, similar occurrences of AEs were observed in both treatment groups in either patients with and without renal impairment. A slight trend towards more AEs was observed in patients with an eGFR <60 mL/min/1.73 m^2^ than those patients with an eGFR ≥60 mL/min/1.73 m^2^. Regarding AEs indicative of renal impairment, more AEs indicating renal impairment were overall reported for patients with renal impairment compared with those without renal impairment. Patients treated with serelaxin experienced fewer renal AEs regardless of eGFR subgroup. In subgroup eGFR <60 mL/min/1.73 m^2^, AEs indicative of renal impairment through day 5 were reported in 6 % of patients treated with serelaxin, compared with 10.8 % of patients treated with placebo. In subgroup eGFR ≥60 mL/min/1.73 m^2^, the proportions were 1.3 % for serelaxin-treated patients and 2.5 % for patients assigned to placebo. Overall, the favorable AE profile observed for serelaxin in RELAX-AHF was maintained in each eGFR category.

**Table 4:** Overview of all-treatment emergent AEs regardless of study drug relationship by eGFR through day 5 and day 14

Adverse event (AE) subset; *n* (%)	eGFR category					
	All patients^a^		eGFR <60 mL/min/1.73 m^2^		eGFR ≥60 mL/min/1.73 m^2^	
	Serelaxin (*N* = 568)	Placebo (*N* = 570)	Serelaxin (*N* = 403)	Placebo (*N* = 406)	Serelaxin (*N* = 155)	Placebo (*N* = 157)
AEs through day 5						
Any AEs	280 (49.3)	305 (53.5)	209 (51.9)	231 (56.9)	66 (42.6)	70 (44.6)
Any AEs leading to drug discontinuation	26 (4.6)	22 (3.9)	16 (4.0)	16 (3.9)	10 (6.5)	5 (3.2)
SAEs	36 (6.3)	38 (6.7)	27 (6.7)	29 (7.1)	8 (5.2)	7 (4.5)
SAEs with an outcome of death	6 (1.1)	9 (1.6)	4 (1.0)	6 (1.5)	2 (1.3)	2 (1.3)
AEs through day 14						
All-treatment emergent AEs	305 (53.7)	320 (56.1)	229 (56.8)	243 (59.9)	70 (45.2)	73 (46.5)
SAEs	86 (15.1)	78 (13.7)	68 (16.9)	58 (14.3)	16 (10.3)	18 (11.5)
SAEs with an outcome of death	10 (1.8)	15 (2.6)	7 (1.7)	10 (2.5)	3 (1.9)	4 (2.5)
AEs indicative of renal impairment through day 5						
Subjects with any AE	26 (4.6)	49 (8.6)	24 (6.0)	44 (10.8)	2 (1.3)	4 (2.5)
Azotaemia	1 (0.2)	1 (0.2)	1 (0.2)	1 (0.2)	–	–
Blood creatinine increased	14 (2.5)	22 (3.9)	12 (3.0)	19 (4.7)	2 (1.3)	3 (1.9)
Oliguria	0	1 (0.2)	–	–	0	1 (0.6)
Proteinuria	0	2 (0.4)	0	2 (0.5)	–	–
Renal failure	9 (1.6)	23 (4.0)	9 (2.2)	21 (5.2)	0	1 (0.6)
Renal failure acute	1 (0.2)	0	1 (0.2)	0	–	–
Renal impairment	1 (0.2)	1 (0.2)	1 (0.2)	1 (0.2)	–	–
AEs indicative of renal impairment through day 14						
Subjects with any AE	32 (5.6)	51 (8.9)	30 (7.4)	46 (11.3)	2 (1.3)	4 (2.5)
Azotaemia	1 (0.2)	1 (0.2)	1 (0.2)	1 (0.2)	–	–
Blood creatinine increased	14 (2.5)	23 (4.0)	12 (3.0)	20 (4.9)	2 (1.3)	3 (1.9)
Oliguria	0	1 (0.2)	–	–	0	1 (0.6)
Proteinuria	0	2 (0.4)	0	2 (0.5)	–	–
Renal failure	14 (2.5)	25 (4.4)	14 (3.5)	23 (5.7)	0	1 (0.6)
Renal failure acute	2 (0.4)	0	2 (0.5)	0	–	–
Renal impairment	1 (0.2)	1 (0.2)	1 (0.2)	1 (0.2)	–	–

^a^eGFR was 30–75 mL/min/1.73 m^2^ according to the inclusion criteria

## Discussion

In the current post hoc analysis of the RELAX-AHF trial, we demonstrate that baseline renal dysfunction (eGFR <60 mL/min/1.73 m^2^) was associated with worse clinical outcomes in acute heart failure patients treated with placebo. Serelaxin appears to attenuate this effect, though this trend was not statistically significant. Moreover, in acute heart failure patients with renal dysfunction, treatment with serelaxin resulted in larger reductions of 60 day CV death and 180 day all-cause mortality compared with patients without renal dysfunction, and a lower NNT compared to the overall study population. Our findings suggest that treatment with serelaxin may have a greater treatment effect in patients with renal dysfunction and that the observed reduction of (cardiovascular) mortality by serelaxin seems to be driven by more pronounced effects in patients with more severe renal dysfunction.

Renal dysfunction is common in patients with acute heart failure and is associated with increased mortality and rehospitalization [1, 2]. In fact, the prognostic value of renal impairment in patients with heart failure remained consistent even across a large number of studies, as was recently demonstrated by Damman and colleagues in a meta-analysis including 1,076,104 heart failure patients [21].

Recent studies demonstrated that both relaxin and its receptor are expressed in the heart and kidney [22–25], suggesting that relaxin may also have direct renal effects. Indeed, previous pre-clinical studies in rats have demonstrated that relaxin causes renal vasodilatation, resulting in increased renal plasma flow and glomerular filtration rate, protecting the kidney against renal ischemia–reperfusion injury, and attenuating renal fibrosis [26–30]. In both healthy humans and patients with chronic stable heart failure, administration of serelaxin resulted in an increased renal blood flow [11, 12]. Furthermore, angiotensin II induced renal vasoconstriction was reversed by relaxin, underscoring the renal protective effects of relaxin [27]. Post hoc analysis of the RELAX-AHF demonstrated that treatment with serelaxin was associated with a lower increase in markers of end-organ damage, including the renal markers creatinine and cystatin C; although serelaxin was administered for 48 h, creatinine levels remained lower through day 5, while cystatine C levels even remained lower through day 14 compared to the placebo group [9]. These observations suggest that serelaxin might possess renal protective properties that may even result in long-term beneficial effects after its administration in acute heart failure patients.

In our study, we found no significant interactions between the association of renal impairment and poor clinical outcome and study treatment. However, a trend was observed suggesting that patients with renal dysfunction treated with placebo had a higher risk for CV mortality and all-cause mortality compared to patients with renal dysfunction treated with serelaxin. Interestingly, the survival curves of patients with renal dysfunction treated with serelaxin were almost comparable with the survival curves of patients with normal renal function, irrespective of their study treatment. When we studied the treatment effect of serelaxin more in depth on other clinical outcomes [31], we found overall higher treatment estimates of serelaxin in the eGFR <60 ml/min/1.73 m^2^ subgroup, compared with the eGFR ≥60 ml/min/1.73 m^2^ subgroup. Although these interactions were not statistically significant, our results suggest that serelaxin may have a greater treatment effect in patients with renal impairment; patients with renal impairment treated with serelaxin had greater dyspnea relief, shorter stay in the hospital, and lower use of IV diuretics. It should be noted that the lower diuretic requirement might also explain the favorable changes in levels of the renal markers. In terms of CV through day 60 and all-cause mortality through day 180, treatment with serelaxin resulted in a greater treatment benefit in patients with an eGFR <60 ml/min/1.73 m^2^ compared with patients with an eGFR ≥60 ml/min/1.73 m^2^. In addition, we found a lower NNT in both the eGFR <60 ml/min/1.73 m^2^ subgroups for CV mortality, as well as all-cause mortality, compared with the overall study population. Because the NNT is the inverse of the absolute risk reduction, the NNT will vary according to the event rates of both subgroups, since it is expected that a higher clinical risk is related with a higher absolute risk reduction. However, we demonstrated that the relative risk reduction by treatment with serelaxin was also not homogeneous among the RELAX-AHF study population. The kidney itself is evidently a target organ for relaxin activity and this may explain why patients with renal impairment may benefit more from the renoprotective effects of serelaxin administration. In addition, as these patients are at higher clinical risk, treatment may have had greater impact in these patients compared to ‘low risk’ patients. Our results illustrate that a patient’s baseline risk may interact with treatment in a clinically relevant manner and that the overall treatment effect may not reflect the treatment effect of the individual patients [32].

In addition to the well-known expected limitations of such post hoc and retrospective analyses, the present study has several limitations. Subgroup analyses are necessary to evaluate potential heterogeneity in treatment effect, but our results demonstrate that these analyses are associated with statistical concerns [33, 34]. The subgroup eGFR ≥60 ml/min/1.73 m^2^ had a smaller sample size and while the estimates of treatment effect of serelaxin were slightly higher in the eGFR <60 ml/min/1.73 m^2^ subgroup, the present study lacks the power to detect treatment interactions between subgroups. In addition, the subgroup eGFR ≥60 ml/min/1.73 m^2^ was relatively small and had lower number of events. The mortality rates for this subgroup were 14/315 = 4.4 % for CV mortality and 18/315 = 5.7 % for all-cause mortality. The small number of patients and events may have limited our analysis and interpretation of the results of this subgroup. One could also suggest that it may have been difficult for any treatment to have an impact in these ‘low risk’ patients. Further, not every patient had eGFR at baseline. Since the RELAX-AHF trial only enrolled patients with renal function 30–75 ml/min/1.73 m^2^, we could not assess the effects of serelaxin of patients with a renal function out of these ranges. As the subgroups were not pre-specified, our results should be interpreted with caution. Cystatin C was measured in the RELAX-AHF trial and is proposed as a better and more reliable marker of renal function as cystatin C is unaffected by increases in age, diet, and muscle mass [35–40]. Unfortunately, cystatin C was not measured in all patients who were enrolled in the RELAX-AHF trial. The number of patients with missing values of cystatin C formed a limitation for the analysis of eGFR estimated by cystatin C. Therefore, we did not include analyses on eGFR estimated by cystatin C in the current paper. It should also be noted that the primary endpoint of the RELAX-AHF trial was dyspnea relief. Other outcomes regarding total dose of diuretic, worsening heart failure, hospital stay, CV death through day 60 and all-cause mortality through day 180 were not among the primary endpoints of the RELAX-AHF trial. Thus, the RELAX-AHF study was not primarily designed to address these endpoints. Currently, the replicate phase III study is ongoing, studying the effect of serelaxin on CV death and other clinical outcomes (RELAX-AHF-2 trial, NCT01870778). Whether treatment with serelaxin results in a greater treatment benefit in patients with an eGFR <60 mL/min/1.73 m^2^ should be further investigated.

In summary, treatment with serelaxin was safe and effective and trends towards an attenuation of the well-known association between renal dysfunction and clinical outcomes were observed in patients treated with serelaxin. Our results suggest that serelaxin has a greater treatment benefit in terms of reducing CV and all-cause mortality in patients with an eGFR <60 mL/min/1.73 m^2^. However, as these were not the primary endpoints of the RELAX-AHF trial, this finding should be interpreted with caution and ongoing and future studies with larger sample sizes are needed to confirm these findings. Our findings may emphasize the safety profile of serelaxin, as these patients are considered to be more vulnerable and at higher clinical risk.

## References

[1] Metra M, Davison B, Bettari L (2012). Is worsening renal function an ominous prognostic sign in patients with acute heart failure? The role of congestion and its interaction with renal function. Circ Heart Fail.

[2] Yancy CW, Lopatin M, Stevenson LW, De Marco T, Fonarow GC, ADHERE Scientific Advisory Committee and Investigators (2006). Clinical presentation, management, and in-hospital outcomes of patients admitted with acute decompensated heart failure with preserved systolic function: a report from the Acute Decompensated Heart Failure National Registry (ADHERE) Database. J Am Coll Cardiol.

[3] Akhter MW, Aronson D, Bitar F (2004). Effect of elevated admission serum creatinine and its worsening on outcome in hospitalized patients with decompensated heart failure. Am J Cardiol.

[4] Metra M, Cotter G, Gheorghiade M, Dei Cas L, Voors AA (2012). The role of the kidney in heart failure. Eur Heart J.

[5] Schmieder RE, Mitrovic V, Hengstenberg C (2015). Renal impairment and worsening of renal function in acute heart failure: can new therapies help? The potential role of serelaxin. Clin Res Cardiol.

[6] Sobotka PA, Mahfoud F, Schlaich MP, Hoppe UC, Bohm M, Krum H (2011). Sympatho-renal axis in chronic disease. Clin Res Cardiol.

[7] Smilde TD, Damman K, van der Harst P (2009). Differential associations between renal function and “modifiable” risk factors in patients with chronic heart failure. Clin Res Cardiol.

[8] Teerlink JR, Cotter G, Davison BA (2013). Serelaxin, recombinant human relaxin-2, for treatment of acute heart failure (RELAX-AHF): a randomised, placebo-controlled trial. Lancet.

[9] Metra M, Cotter G, Davison BA (2013). Effect of serelaxin on cardiac, renal, and hepatic biomarkers in the Relaxin in Acute Heart Failure (RELAX-AHF) development program: correlation with outcomes. J Am Coll Cardiol.

[10] Khanna D, Clements PJ, Furst DE (2009). Recombinant human relaxin in the treatment of systemic sclerosis with diffuse cutaneous involvement: a randomized, double-blind, placebo-controlled trial. Arthritis Rheum.

[11] Smith MC, Danielson LA, Conrad KP, Davison JM (2006). Influence of recombinant human relaxin on renal hemodynamics in healthy volunteers. J Am Soc Nephrol.

[12] Voors AA, Dahlke M, Meyer S (2014). Renal hemodynamic effects of serelaxin in patients with chronic heart failure: a randomized, placebo-controlled study. Circ Heart Fail.

[13] Ponikowski P, Mitrovic V, Ruda M (2014). A randomized, double-blind, placebo-controlled, multicentre study to assess haemodynamic effects of serelaxin in patients with acute heart failure. Eur Heart J.

[14] Metra M, Ponikowski P, Cotter G (2013). Effects of serelaxin in subgroups of patients with acute heart failure: results from RELAX-AHF. Eur Heart J.

[15] Schmieder RE (2013). RELAX-AHF: consistency across subgroups and new hypotheses generated. Eur Heart J.

[16] Ponikowski P, Metra M, Teerlink JR (2012). Design of the RELAXin in acute heart failure study. Am Heart J.

[17] Levey AS, Bosch JP, Lewis JB, Greene T, Rogers N, Roth D (1999). A more accurate method to estimate glomerular filtration rate from serum creatinine: a new prediction equation. Modification of Diet in Renal Disease Study Group. Ann Intern Med.

[18] Levey AS, Greene T, Kusek JW, Beck GJ, Group MS (2000). A simplified equation to predict glomerular filtration rate from serum creatinine [Abstract]. Am Soc Nephrol.

[19] Inker LA, Schmid CH, Tighiouart H (2012). Estimating glomerular filtration rate from serum creatinine and cystatin C. N Engl J Med.

[20] Altman DG, Andersen PK (1999). Calculating the number needed to treat for trials where the outcome is time to an event. BMJ.

[21] Damman K, Valente MA, Voors AA, O’Connor CM, van Veldhuisen DJ, Hillege HL (2014). Renal impairment, worsening renal function, and outcome in patients with heart failure: an updated meta-analysis. Eur Heart J.

[22] Dschietzig T, Richter C, Bartsch C (2001). The pregnancy hormone relaxin is a player in human heart failure. FASEB J.

[23] Samuel CS, Hewitson TD (2009). Relaxin and the progression of kidney disease. Curr Opin Nephrol Hypertens.

[24] Hsu SY, Nakabayashi K, Nishi S (2002). Activation of orphan receptors by the hormone relaxin. Science.

[25] Cernaro V, Lacquaniti A, Lupica R (2014). Relaxin: new pathophysiological aspects and pharmacological perspectives for an old protein. Med Res Rev.

[26] Danielson LA, Conrad KP (2003). Time course and dose response of relaxin-mediated renal vasodilation, hyperfiltration, and changes in plasma osmolality in conscious rats. J Appl Physiol (1985).

[27] Danielson LA, Sherwood OD, Conrad KP (1999). Relaxin is a potent renal vasodilator in conscious rats. J Clin Invest.

[28] Collino M, Rogazzo M, Pini A (2013). Acute treatment with relaxin protects the kidney against ischaemia/reperfusion injury. J Cell Mol Med.

[29] Bogzil AH, Ashton N (2009). Relaxin-induced changes in renal function and RXFP1 receptor expression in the female rat. Ann N Y Acad Sci.

[30] Bogzil AH, Eardley R, Ashton N (2005). Relaxin-induced changes in renal sodium excretion in the anesthetized male rat. Am J Physiol Regul Integr Comp Physiol.

[31] Felker GM, Teerlink JR, Butler J (2014). Effect of serelaxin on mode of death in acute heart failure: results from the RELAX-AHF study. J Am Coll Cardiol.

[32] Ferreira JP, Santos M, Almeida S, Marques I, Bettencourt P, Carvalho H (2013). Tailoring diuretic therapy in acute heart failure: insight into early diuretic response predictors. Clin Res Cardiol.

[33] Brookes ST, Whitely E, Egger M, Smith GD, Mulheran PA, Peters TJ (2004). Subgroup analyses in randomized trials: risks of subgroup-specific analyses; power and sample size for the interaction test. J Clin Epidemiol.

[34] Schulz KF, Grimes DA (2005). Multiplicity in randomised trials II: subgroup and interim analyses. Lancet.

[35] Dharnidharka VR, Kwon C, Stevens G (2002). Serum cystatin C is superior to serum creatinine as a marker of kidney function: a meta-analysis. Am J Kidney Dis.

[36] Damman K, van der Harst P, Smilde TD (2012). Use of cystatin C levels in estimating renal function and prognosis in patients with chronic systolic heart failure. Heart.

[37] Laterza OF, Price CP, Scott MG (2002). Cystatin C: an improved estimator of glomerular filtration rate?. Clin Chem.

[38] Coll E, Botey A, Alvarez L (2000). Serum cystatin C as a new marker for noninvasive estimation of glomerular filtration rate and as a marker for early renal impairment. Am J Kidney Dis.

[39] Hojs R, Bevc S, Ekart R, Gorenjak M, Puklavec L (2008). Serum cystatin C-based equation compared to serum creatinine-based equations for estimation of glomerular filtration rate in patients with chronic kidney disease. Clin Nephrol.

[40] Valente MA, Hillege HL, Navis G (2014). The Chronic Kidney Disease Epidemiology Collaboration equation outperforms the Modification of Diet in Renal Disease equation for estimating glomerular filtration rate in chronic systolic heart failure. Eur J Heart Fail.

